# 2,2′-Diamino-4,4′-bi-1,3-thia­zolium bis­(3-nitro­benzoate)

**DOI:** 10.1107/S1600536809005856

**Published:** 2009-02-25

**Authors:** Bing-Xin Liu, Yan-Ping Yu, Yuan-Yuan Lin, Mei Du

**Affiliations:** a Department of Chemistry, Shanghai University, People’s Republic of China

## Abstract

In the title salt, C_6_H_8_N_4_S_2_^2+^·2C_7_H_4_NO_4_^−^, the diprotonated diamino­bithia­zole dication is located on an inversion center. The carboxyl­ate group of the anion is twisted with respect to the benzene ring, with a dihedral angle of 13.6 (4)°. N—H⋯O hydrogen bonds involving the amino and ammonium groups of the dication and the carboxyl­ate functionality of the anion generate supra­molecular chains in the crystal.

## Related literature

For applications of complexes including 2,2′-diamino-4,4′-bi-1,3-thia­zole as ligand, see: Sun *et al.* (1997 ▶); Waring (1981 ▶); Fisher *et al.* (1985 ▶). For related structures, see: Liu *et al.* (2003 ▶, 2005 ▶).
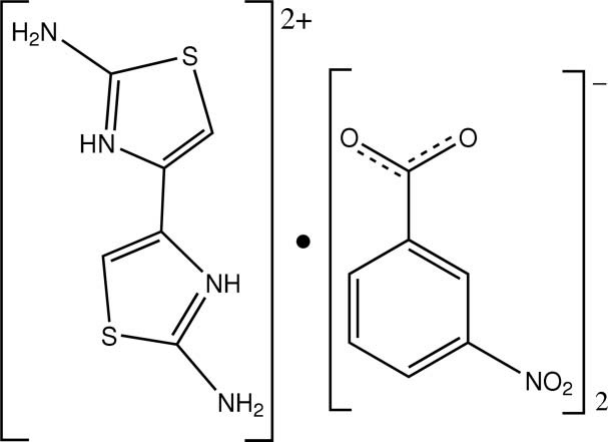


## Experimental

### 

#### Crystal data

C_6_H_8_N_4_S_2_^2+^·2C_7_H_4_NO_4_^−^
*M* *_r_* = 532.51
Triclinic, 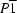

*a* = 6.5670 (13) Å
*b* = 7.4538 (15) Å
*c* = 12.301 (2) Å
α = 74.747 (2)°
β = 89.721 (2)°
γ = 70.483 (2)°
*V* = 545.26 (19) Å^3^
*Z* = 1
Mo *K*α radiation
μ = 0.31 mm^−1^
*T* = 295 K
0.20 × 0.18 × 0.15 mm

#### Data collection

Bruker APEXII diffractometer
Absorption correction: multi-scan (*ABSCOR*; Higashi, 1995 ▶) *T*_min_ = 0.940, *T*_max_ = 0.955
2771 measured reflections
1880 independent reflections
1325 reflections with *I* > 2σ(*I*)
*R*_int_ = 0.018

#### Refinement

*R*[*F* ^2^ > 2σ(*F* ^2^)] = 0.051
*wR*(*F* ^2^) = 0.140
*S* = 1.08
1880 reflections
164 parameters
H-atom parameters constrained
Δρ_max_ = 0.24 e Å^−3^
Δρ_min_ = −0.35 e Å^−3^

### 

Data collection: *APEX2* (Bruker, 2004 ▶); cell refinement: *SAINT* (Bruker, 2004 ▶); data reduction: *SAINT*; program(s) used to solve structure: *SIR92* (Altomare *et al.*, 1993 ▶); program(s) used to refine structure: *SHELXL97* (Sheldrick, 2008 ▶); molecular graphics: *ORTEP-3 for Windows* (Farrugia, 1997 ▶); software used to prepare material for publication: *WinGX* (Farrugia, 1999 ▶).

## Supplementary Material

Crystal structure: contains datablocks I, global. DOI: 10.1107/S1600536809005856/bh2208sup1.cif

Structure factors: contains datablocks I. DOI: 10.1107/S1600536809005856/bh2208Isup2.hkl

Additional supplementary materials:  crystallographic information; 3D view; checkCIF report

## Figures and Tables

**Table 1:** Hydrogen-bond geometry (Å, °)

*D*—H⋯*A*	*D*—H	H⋯*A*	*D*⋯*A*	*D*—H⋯*A*
N11—H11*A*⋯O22	0.95	1.71	2.627 (4)	161
N12—H12*B*⋯O21	0.92	1.86	2.770 (4)	171
N12—H12*A*⋯O21^i^	0.90	2.08	2.830 (4)	140

Symmetry code: (i) 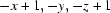.

**Fig. 1.**
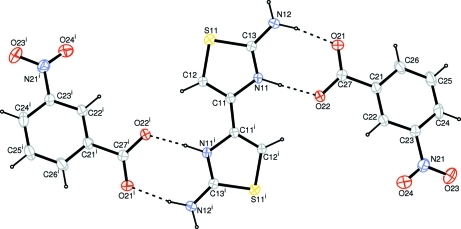
The molecular structure of the title salt, with 30% probability displacement ellipsoids (arbitrary spheres for H atoms), dashed lines showing the hydrogen bonding within the complex [symmetry code: (i) 2 - x,1 - y,1 - z].

**Fig. 2.**
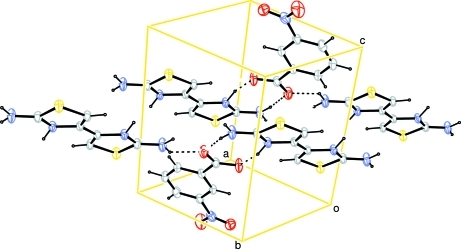
The unit cell packing diagram showing hydrogen bondings between thiazole rings and carboxyl groups of 3-nitrobenzoate anions.

## supplementary crystallographic information

### Comment

Transition metal complexes of 2,2'-diamino-4,4'-bi-1,3-thiazole (DABT) have shown potential applications in the field of soft magnetic material (Sun *et al.*, 1997) and biological activities, such as the effective inhibition of DNA synthesis in tumor cells (Waring, 1981; Fisher *et al.*, 1985). As part of a serial structural investigation of metal complexes with DABT (Liu *et al.*, 2003), while preparing the Mn^II^ complex of DABT, the title H_2_DABT^2+^ salt was unexpectedly obtained, and its X-ray structure is presented here.

The structure of the title salt is shown in Fig. 1. The diprotonated DABT dication, H_2_DABT, is located on an inversion center while the 3-nitrobenzoate anion is placed in general position. The H_2_DABT moiety displays a *trans* planar configuration, which agrees with that found in the neutral DABT (Liu *et al.*, 2003). The *C*—*N*(amino) bond length of 1.333 (4) Å is similar to the *C*—*N*(thiazole ring) bond length, 1.316 (4) Å, indicating electron delocalization between the amino and thiazole groups. It is notable that the protonated N atoms form hydrogen bonds to O atoms of carboxylate groups of 3-nitrobenzoate anions, to form supramolecular chains. This feature is consistent with those found in the H_2_DABT salt formed with 2-nitrobenzoate, which we reported previously (Liu *et al.*, 2005).

The carboxylate group of 3-nitrobenzoate anion is twisted with respect to the benzene plane, with a dihedral angle of 13.6 (4)°, which is comparable with 13.1 (2)° found in the 2-nitrobenzoate H_2_DABT salt (Liu *et al.*, 2005). This distortion allows the formation of the observed supramolecular structure (Fig. 2).

### Experimental

An ethanol-water solution (1:1, 30 ml) of DABT (0.20 g, 1 mmol) and MnCl_2_.4H_2_O (0.20 g, 1 mmol) was mixed with another aqueous solution (10 ml) of 3-nitrobenzoic acid (0.33 g, 2 mmol) and NaOH (0.08 g, 2 mmol). The mixture was refluxed for 6 h. After cooling to room temperature, the solution was filtered. Yellow single crystals were obtained from the filtrate after 5 d.

### Refinement

H atoms bonded to C atoms were placed in calculated positions with C—H = 0.93 Å, and included in the final cycles of refinement in riding mode, with *U*_iso_(H) = 1.2*U*_eq_(carrier C). H atoms bonded to N atoms were located in a difference map and refined as riding in their as found relative positions, with *U*_iso_(H) = 1.2*U*_eq_(carrier N).

### Crystal data

**Table:**

C_6_H_8_N_4_S_2_^2+^·2C_7_H_4_NO_4_^−^	*Z* = 1
*M**_r_* = 532.51	*F*(000) = 274
Triclinic, *P*1	*D*_x_ = 1.622 Mg m^−^^3^
Hall symbol: -P 1	Mo *K*α radiation, λ = 0.71073 Å
*a* = 6.5670 (13) Å	Cell parameters from 1780 reflections
*b* = 7.4538 (15) Å	θ = 2.0–25.0°
*c* = 12.301 (2) Å	µ = 0.31 mm^−^^1^
α = 74.747 (2)°	*T* = 295 K
β = 89.721 (2)°	Prism, yellow
γ = 70.483 (2)°	0.20 × 0.18 × 0.15 mm
*V* = 545.26 (19) Å^3^	

### Data collection

**Table:**

Bruker APEXII diffractometer	1880 independent reflections
Radiation source: fine-focus sealed tube	1325 reflections with *I* > 2σ(*I*)
graphite	*R*_int_ = 0.018
Detector resolution: 10.0 pixels mm^-1^	θ_max_ = 25.0°, θ_min_ = 3.0°
ω scans	*h* = −7→7
Absorption correction: multi-scan (*ABSCOR*; Higashi, 1995)	*k* = −8→8
*T*_min_ = 0.940, *T*_max_ = 0.955	*l* = −14→12
2771 measured reflections	

### Refinement

**Table:**

Refinement on *F*^2^	Secondary atom site location: difference Fourier map
Least-squares matrix: full	Hydrogen site location: inferred from neighbouring sites
*R*[*F*^2^ > 2σ(*F*^2^)] = 0.051	H-atom parameters constrained
*wR*(*F*^2^) = 0.140	*w* = 1/[σ^2^(*F*_o_^2^) + (0.0733*P*)^2^ + 0.0511*P*] where *P* = (*F*_o_^2^ + 2*F*_c_^2^)/3
*S* = 1.08	(Δ/σ)_max_ < 0.001
1880 reflections	Δρ_max_ = 0.24 e Å^−^^3^
164 parameters	Δρ_min_ = −0.35 e Å^−^^3^
0 restraints	Extinction correction: *SHELXL97* (Sheldrick, 2008), Fc^*^=kFc[1+0.001xFc^2^λ^3^/sin(2θ)]^-1/4^
0 constraints	Extinction coefficient: 0.013 (5)
Primary atom site location: structure-invariant direct methods	

### Fractional atomic coordinates and isotropic or equivalent isotropic displacement parameters (Å^2^)

**Table:**

	*x*	*y*	*z*	*U*_iso_*/*U*_eq_	
N11	0.8309 (4)	0.3447 (4)	0.4956 (2)	0.0409 (7)	
H11A	0.7922	0.3044	0.5705	0.049*	
N12	0.6759 (5)	0.1415 (4)	0.4388 (2)	0.0573 (8)	
H12A	0.6577	0.0819	0.3869	0.069*	
H12B	0.5903	0.1378	0.4984	0.069*	
S11	0.97982 (15)	0.25520 (13)	0.31830 (7)	0.0483 (3)	
C12	1.0789 (5)	0.4030 (5)	0.3738 (2)	0.0398 (8)	
H12	1.1856	0.4523	0.3432	0.048*	
C11	0.9837 (5)	0.4382 (4)	0.4663 (2)	0.0358 (7)	
C13	0.8101 (5)	0.2415 (5)	0.4254 (3)	0.0422 (8)	
O21	0.4454 (4)	0.1470 (3)	0.62785 (19)	0.0555 (7)	
O22	0.6468 (4)	0.2977 (4)	0.6861 (2)	0.0613 (7)	
O23	0.3437 (6)	0.3196 (5)	1.1652 (2)	0.0933 (11)	
O24	0.6375 (4)	0.2992 (4)	1.0836 (2)	0.0616 (7)	
N21	0.4533 (6)	0.2964 (4)	1.0853 (2)	0.0539 (8)	
C21	0.3836 (5)	0.2210 (4)	0.8031 (3)	0.0386 (7)	
C22	0.4661 (5)	0.2654 (4)	0.8919 (3)	0.0395 (8)	
H22	0.5923	0.2963	0.8868	0.047*	
C23	0.3564 (5)	0.2627 (4)	0.9888 (3)	0.0419 (8)	
C24	0.1652 (6)	0.2268 (5)	0.9973 (3)	0.0537 (9)	
H24	0.0916	0.2314	1.0618	0.064*	
C25	0.0836 (6)	0.1837 (5)	0.9081 (3)	0.0561 (10)	
H25	−0.0461	0.1585	0.9124	0.067*	
C26	0.1939 (5)	0.1776 (5)	0.8120 (3)	0.0474 (8)	
H26	0.1403	0.1441	0.7532	0.057*	
C27	0.5007 (5)	0.2205 (5)	0.6972 (3)	0.0429 (8)	

### Atomic displacement parameters (Å^2^)

**Table:**

	*U*^11^	*U*^22^	*U*^33^	*U*^12^	*U*^13^	*U*^23^
N11	0.0525 (17)	0.0566 (16)	0.0301 (14)	−0.0364 (14)	0.0145 (12)	−0.0167 (13)
N12	0.078 (2)	0.081 (2)	0.0511 (18)	−0.0614 (18)	0.0273 (15)	−0.0379 (16)
S11	0.0631 (6)	0.0653 (6)	0.0383 (5)	−0.0408 (5)	0.0183 (4)	−0.0267 (4)
C12	0.0464 (19)	0.0569 (19)	0.0322 (17)	−0.0337 (16)	0.0140 (14)	−0.0186 (15)
C11	0.0422 (18)	0.0414 (17)	0.0304 (17)	−0.0233 (15)	0.0065 (13)	−0.0092 (14)
C13	0.051 (2)	0.0550 (19)	0.0349 (18)	−0.0319 (17)	0.0131 (15)	−0.0186 (16)
O21	0.0740 (17)	0.0766 (16)	0.0456 (14)	−0.0524 (14)	0.0194 (12)	−0.0322 (13)
O22	0.0789 (18)	0.0976 (19)	0.0507 (15)	−0.0702 (16)	0.0340 (13)	−0.0407 (14)
O23	0.124 (3)	0.138 (3)	0.0601 (19)	−0.078 (2)	0.0518 (18)	−0.057 (2)
O24	0.0703 (18)	0.0725 (17)	0.0531 (16)	−0.0340 (15)	0.0039 (13)	−0.0236 (13)
N21	0.076 (2)	0.0540 (18)	0.0406 (18)	−0.0319 (17)	0.0166 (16)	−0.0155 (14)
C21	0.0428 (19)	0.0377 (17)	0.0396 (18)	−0.0201 (15)	0.0107 (14)	−0.0099 (14)
C22	0.0401 (18)	0.0433 (17)	0.0429 (19)	−0.0228 (15)	0.0125 (14)	−0.0142 (15)
C23	0.0459 (19)	0.0469 (18)	0.0389 (19)	−0.0227 (16)	0.0132 (15)	−0.0132 (15)
C24	0.050 (2)	0.061 (2)	0.054 (2)	−0.0255 (19)	0.0257 (17)	−0.0140 (18)
C25	0.041 (2)	0.069 (2)	0.062 (3)	−0.0305 (19)	0.0158 (18)	−0.011 (2)
C26	0.043 (2)	0.053 (2)	0.051 (2)	−0.0274 (17)	0.0062 (16)	−0.0096 (17)
C27	0.051 (2)	0.0515 (19)	0.0365 (18)	−0.0282 (17)	0.0112 (15)	−0.0157 (16)

### Geometric parameters (Å, °)

**Table:**

N11—C13	1.333 (4)	O24—N21	1.217 (4)
N11—C11	1.398 (4)	N21—C23	1.466 (4)
N11—H11A	0.9526	C21—C22	1.383 (4)
N12—C13	1.316 (4)	C21—C26	1.384 (4)
N12—H12A	0.8973	C21—C27	1.510 (4)
N12—H12B	0.9215	C22—C23	1.388 (4)
S11—C13	1.726 (3)	C22—H22	0.9300
S11—C12	1.727 (3)	C23—C24	1.367 (5)
C12—C11	1.339 (4)	C24—C25	1.378 (5)
C12—H12	0.9300	C24—H24	0.9300
C11—C11^i^	1.456 (5)	C25—C26	1.386 (5)
O21—C27	1.242 (4)	C25—H25	0.9300
O22—C27	1.263 (4)	C26—H26	0.9300
O23—N21	1.230 (4)		
			
C13—N11—C11	113.5 (2)	C22—C21—C27	119.8 (3)
C13—N11—H11A	116.6	C26—C21—C27	120.5 (3)
C11—N11—H11A	124.5	C21—C22—C23	118.6 (3)
C13—N12—H12A	120.5	C21—C22—H22	120.7
C13—N12—H12B	121.6	C23—C22—H22	120.7
H12A—N12—H12B	117.7	C24—C23—C22	122.3 (3)
C13—S11—C12	90.11 (14)	C24—C23—N21	119.6 (3)
C11—C12—S11	111.9 (2)	C22—C23—N21	118.1 (3)
C11—C12—H12	124.0	C23—C24—C25	118.6 (3)
S11—C12—H12	124.0	C23—C24—H24	120.7
C12—C11—N11	112.7 (3)	C25—C24—H24	120.7
C12—C11—C11^i^	128.3 (3)	C24—C25—C26	120.4 (3)
N11—C11—C11^i^	119.0 (3)	C24—C25—H25	119.8
N12—C13—N11	122.9 (3)	C26—C25—H25	119.8
N12—C13—S11	125.3 (2)	C21—C26—C25	120.3 (3)
N11—C13—S11	111.8 (2)	C21—C26—H26	119.8
O24—N21—O23	122.9 (3)	C25—C26—H26	119.8
O24—N21—C23	119.1 (3)	O21—C27—O22	125.3 (3)
O23—N21—C23	118.0 (3)	O21—C27—C21	118.0 (3)
C22—C21—C26	119.7 (3)	O22—C27—C21	116.7 (3)

Symmetry codes: (i) −*x*+2, −*y*+1, −*z*+1.

### Hydrogen-bond geometry (Å, °)

**Table:**

*D*—H···*A*	*D*—H	H···*A*	*D*···*A*	*D*—H···*A*
N11—H11A···O22	0.95	1.71	2.627 (4)	161
N12—H12B···O21	0.92	1.86	2.770 (4)	171
N12—H12A···O21^ii^	0.90	2.08	2.830 (4)	140

Symmetry codes: (ii) −*x*+1, −*y*, −*z*+1.
